# Genomic Characterization of a *Mycoplasma ovipneumoniae* Strain from Hu Sheep in Inner Mongolia, China

**DOI:** 10.3390/vetsci13010079

**Published:** 2026-01-13

**Authors:** Lingli Dai, Na Wang, Fan Zhang, Yuemei Zhang, Yue Song, Wei Liu, Xiaodong Cao, Jingyu Shi, Shihua Zhao, Fan Bai

**Affiliations:** 1 Veterinary Research Institute, Inner Mongolia Academy of Agricultural and Animal Husbandry Sciences, Hohhot 010031, China; 2 School of Pharmacy New Drug Safety Evaluation Research Center, Inner Mongolia Medical University, Hohhot 010107, China

**Keywords:** *Mycoplasma ovipneumoniae*, genome sequencing, comparative genomics, Hu sheep, pulmonary adenomatous-like lesions

## Abstract

*Mycoplasma ovipneumoniae* poses a major threat to sheep respiratory health, contributing to significant economic losses in farming communities. In this study, we isolated a novel strain, IM-DMQ, from a Hu sheep in Inner Mongolia that exhibited pulmonary adenomatous-like lesions, which is an uncommon pathological manifestation for this pathogen. The complete genome was sequenced using a hybrid Nanopore and Illumina approach, revealing a 1,039,804 bp circular chromosome with a GC content of 29.15%, encoding 1529 genes. Functional annotation highlighted genes involved in essential metabolic processes and potential virulence mechanisms. Comparative genomic analysis demonstrated that IM-DMQ shares the closest ancestry (ANI: 98.3%) with the Chinese strain NXNK2203, while structural variations and 14 unique genes distinguished it from other global strains. Furthermore, microbial community profiling of the original lung tissue revealed a co-infection background involving multiple bacterial pathogens, offering an etiological context for the severe disease presentation. These results provide the first complete genomic resource for an *M. ovipneumoniae* strain from Inner Mongolia associated with unusual pulmonary pathology, offering insights into its genetic diversity and supporting the future development of targeted diagnostics and vaccines for regional disease control.

## Simple Summary

Sheep farming is a vital industry in Inner Mongolia, but respiratory diseases often threaten flock health and farmers’ livelihoods. In this study, we investigated a specific bacterium (*Mycoplasma ovipneumoniae*) that causes pneumonia in sheep. We isolated a new strain, named IM-DMQ, from a sheep that had unusual tumor-like changes in its lungs, which is a condition not typically linked to this infection. After decoding its complete genetic material, we discovered this strain is genetically most similar to others found in China. We also identified specific genes that may help the bacteria survive and cause disease. These findings provide an important foundation for developing better detection methods and vaccines, which could help protect sheep health and support sustainable farming in the region.

## 1. Introduction

Small ruminant production plays a vital role in sustaining the agricultural economy and maintaining ecological balance in pastoral regions of Inner Mongolia, China. Respiratory diseases represent one of the most significant health challenges in these production systems, causing substantial economic losses and impairing animal welfare. Among the various respiratory pathogens, *Mycoplasma ovipneumoniae* has been identified as a primary etiological agent responsible for chronic pneumonia in sheep and goats [1,2]. Infections typically manifest as persistent coughing, mucopurulent nasal discharge, and growth retardation, often exacerbated by secondary bacterial invasions.

As a member of the Mollicutes class, *M. ovipneumoniae* possesses several distinctive biological characteristics, including a reduced genome size and the absence of a cell wall [3]. These features reflect its evolutionary adaptation to a host-associated lifestyle and its reliance on host-derived nutrients, though it can be cultured in artificial media [4]. It is also important to note that pneumonia caused by *M. ovipneumoniae* is frequently polymicrobial, with secondary bacterial infections significantly contributing to disease severity and progression [1,2]. The pathogenic mechanisms of *M. ovipneumoniae* remain incompletely elucidated, though current understanding suggests a process initiated by adhesion to respiratory epithelium, followed by the induction of inflammatory responses and disruption of mucociliary clearance [5,6]. While the bacterium is widely recognized as a respiratory pathogen, its potential involvement in more severe pulmonary conditions, particularly proliferative lesions, has received limited scientific attention. Emerging evidence from human and veterinary medicine has suggested possible connections between chronic mycoplasma infections and neoplastic development, potentially mediated through mechanisms involving sustained inflammation, genomic instability, and apoptosis dysregulation [7,8,9,10]. However, these potential associations remain subject to scientific debate and require systematic investigation in ovine models.

The advent of high-throughput sequencing technologies has fundamentally trans-formed our capacity to investigate bacterial pathogenesis and evolutionary biology [11]. Whole-genome sequencing, in particular, has enabled comprehensive comparative analyses that reveal crucial insights into genetic diversity, virulence determinants, and population dynamics of bacterial pathogens [12]. To date, several *M. ovipneumoniae* genomes from diverse geographical origins—including Australia, Iceland, and Austria—have been sequenced and made publicly available [13,14]. These genomic resources have begun to unravel the genetic underpinnings of host adaptation and pathogenic strategies. Nevertheless, genomic data concerning Chinese isolates, especially those from the ecologically unique Inner Mongolia region, remain strikingly limited [15,16]. This knowledge gap constrains our understanding of local epidemiology and impedes the development of tailored intervention strategies.

To address these limitations, we designed this study to isolate and genomically characterize a local strain of *M. ovipneumoniae* from a clinical case in Hu sheep, and to contextualize this isolate by profiling the microbial community within the infected lung tissue. We report the successful isolation, phenotypic identification, and complete genome sequencing of strain IM-DMQ alongside an analysis of the polymicrobial background via 16S rRNA gene sequencing, obtained from a sheep presenting pulmonary adenomatous-like lesions. Our investigation included comprehensive genomic annotation, functional analysis, and phylogenetic comparison with globally distributed strains. The results provide the first complete genomic resource for an *M. ovipneumoniae* strain from Inner Mongolia associated with unusual pulmonary pathology, establishing a crucial foundation for future research into its pathogenic mechanisms and facilitating the development of region-specific disease control measures.

## 2. Materials and Methods

### 2.1. Clinical Case Description and Sample Collection

A 10-month-old Hu sheep from a large-scale intensive farm in Inner Mongolia died unexpectedly after exhibiting respiratory symptoms including chronic coughing, nasal discharge, and dyspnea. Postmortem examination was immediately performed, and lung tissue samples exhibiting characteristic lesions were collected under sterile conditions. Tissue specimens were divided into two portions: one preserved in 10% neutral buffered formalin for histopathological examination, and the other stored at −80 °C for pathogen isolation.

### 2.2. Histopathological Analysis

Formalin-fixed lung tissues were processed through standard dehydration and embedding procedures. Serial sections of 4-μm thickness were prepared and stained with hematoxylin and eosin (H&E) following conventional protocols [17]. Histopathological evaluation was conducted using a Nikon Eclipse E100 light microscope(Nikon Corporation, Tokyo, Japan), and digital images were captured with an attached DS-Fi3 camera system.

### 2.3. Bacterial Isolation and Culture

The lung tissue samples from lesion margins were aseptically transferred to a biological safety cabinet, minced into 1-mm^3^ pieces, and homogenized in sterile PBS containing 500 U/mL penicillin. After three washing cycles with fresh PBS, the tissue homogenate was centrifuged at 5000× *g* for 10 min. The supernatant was filtered through 0.45-μm membranes and inoculated into modified KM2 medium at a 1:9 ratio. The modified KM2 medium composition included: RPMI 1640 (500 mL/L), Hanks buffer with 1.7% hydrolyzed lactic protein (300 mL/L), horse serum (200 mL/L), 25% yeast extract (20 mL/L), and 1% thallium acetate (5 mL/L). For solid medium preparation, 1.2% agar powder was added. Cultures were incubated at 37 °C with continuous shaking at 150 rpm for 48 h. Following primary isolation from lung tissue, the bacterial culture was streaked onto solid modified KM2 medium. After 36 h of incubation, a single pinpoint colony was aseptically picked and inoculated into liquid KM2 medium for expansion. After 36 h of growth, the liquid culture was again streaked onto solid medium to obtain isolated colonies. This cycle of single-colony isolation followed by liquid culture expansion was repeated twice, resulting in three successive clonal purification passages. The resulting pure culture, originating from a single progenitor colony, was designated strain IM-DMQ. Prior to preservation, positive isolates underwent three consecutive subcultures, and the final clonal preparation was used for all subsequent genomic sequencing and functional analyses.

### 2.4. Molecular Identification

Presumptive *M. ovipneumoniae* isolates were identified by PCR amplification using primers MO-F (5′-ACGGAATATGTTAGCTT-3′) and MO-R (5′-TTCATCCTGCACTCTGT-3′), which were designed in this study to specifically target a 355-bp region within the 16S rRNA gene of *M. ovipneumoniae*. The PCR reaction system consisted of 2× Taq PCR Mix (25 μL), upstream and downstream primers (10 pmol each), DNA template (1 μL), and ddH_2_O to a final volume of 50 μL. Amplification conditions included: initial denaturation at 94 °C for 5 min; 35 cycles of denaturation at 94 °C for 30 s, annealing at 55 °C for 30 s, and extension at 72 °C for 90 s; with a final extension at 72 °C for 10 min.

### 2.5. Genomic DNA Extraction and Sequencing

Bacterial cells were harvested by centrifugation at 10,000× *g* for 30 min at 4 °C, followed by two washes with PBS. Genomic DNA was extracted using the HiPure Bacterial DNA Kit according to the manufacturer’s protocol for Gram-negative bacteria. DNA quality was verified by 1.0% agarose gel electrophoresis, and quantification was performed using both NanoDrop One spectrophotometer (Thermo Fisher Scientific, Wilmington, DE, USA) and Qubit 3.0 Fluorometer (Thermo Fisher Scientific (Invitrogen brand), Carlsbad, CA, USA).

For comprehensive genome sequencing, a hybrid approach combining Oxford Nanopore Technologies (ONT) and Illumina platforms was employed. ONT library preparation utilized SQK-LSK10 and EXP-NBD104/114 kits (Oxford Nanopore Technologies, Oxford, UK), with sequencing conducted on the PromethION platform. Illumina sequencing libraries were prepared through random fragmentation using Covaris ultrasonication, followed by end repair, A-tailing, adapter ligation, and PCR amplification. Library quality assessment included fragment size analysis using Agilent 2100 Bioanalyzer (Agilent Technologies, Santa Clara, CA, USA) and quantification via Q-PCR. Final sequencing was performed on the Illumina NovaSeq 6000 platform (Illumina, Inc., San Diego, CA, USA) with PE150 configuration.

### 2.6. Genome Assembly and Annotation

The complete genome was assembled using a hybrid approach: GUPPY (v5.0.16) processed Nanopore reads for initial assembly, Fastp (v0.23.2) [18] filtered Illumina reads, and Unicycler (v0.5.0) [19] integrated both datasets for final genome polishing. The hybrid assembly produced a final genome comprising two circular contigs totaling 1,041,300 bp, with the main chromosome being 1,039,804 bp (GC 29.15%) and a smaller 1496-bp contig (GC 26.47%). Genome completeness and contamination were assessed using CheckM v1.2.0 [20] with the lineage-specific workflow for Mycoplasmataceae, yielding an estimated completeness of >90% and contamination of <1%, indicating a high-quality, near-complete assembly. Detailed statistics are in Supplementary Table S2. Genome annotation was performed using Prokka (v1.14.6) [21] for coding sequences, Pseudofinder (v1.1.0) [22] for pseudogene identification, and specialized tools for specific features: MinCED (v0.4.2) for CRISPR arrays, IslandPath-DIMOB (v1.0.6) for genomic islands, PhiSpy (v4.2.21) for prophage regions, and RepeatMasker (v4.1.2-p1) for repetitive elements.

### 2.7. Gene Function Analysis

To elucidate the biological significance of the IM-DMQ genome, we performed multi-level functional characterization of all predicted protein-coding sequences. Initial functional predictions were established through homology-based searches against the NCBI non-redundant (NR) and Reference Sequence (RefSeq) databases using BLAST+ (version 2.11.0) with a significance threshold of E-value < 1 × 10^−5^. Conserved protein domains and family affiliations were identified through hidden Markov model scans against the Pfam [23] and TIGERFAMs databases [24] implemented in HMMER (version 3.3.2) [25].

Metabolic capability assessment was conducted via systematic mapping to the Kyoto Encyclopedia of Genes and Genomes (KEGG) database [26], with special attention to pathways involved in energy acquisition, nutrient transport, and biosynthesis. Functional categorization was further refined through Gene Ontology (GO) term [27] assignment based on high-confidence matches to the manually curated UniProtKB/Swiss-Prot database [28]. Evolutionary relationships and core functional conservation were examined by assignment to Clusters of Orthologous Groups (COG) [29] categories.

Specialized functional analyses included: identification of membrane transport systems through Diamond blastp searches against the Transporter Classification Database (TCDB; E-value < 1 × 10^−5^); prediction of virulence determinants via the Virulence Factor Database (VFDB); and annotation of pathogen-host interaction factors using the PHI database with optimized search parameters.

### 2.8. Comparative Genomic Analysis

For comparative genomic analysis, publicly available complete genome sequences of *M. ovipneumoniae* were selected to represent strains from distinct geographic origins (Australia, Iceland, Austria, and China). Average Nucleotide Identity (ANI) values between IM-DMQ and reference strains were calculated using FastANI (v1.34) (https://github.com/ParBLiSS/FastANI, accessed on 30 January 2025). Genome collinearity analysis and visualization were performed with TBtools-II (version 2.138) [30] and Python (version 3.13) scripts. Virulence factors were annotated against the Virulence Factor Database (VFDB), and pathogen-host interactions were analyzed using the PHI database.

### 2.9. Analysis of the Microbial Community in Sheep Lung Tissue Samples by 16S rRNA Gene Sequencing

To comprehensively characterize the microbial community in the lungs of deceased sheep, total DNA was extracted from preserved lung tissue samples (the same samples used for bacterial isolation) using the DNeasy Blood & Tissue Kit (QIAGEN), following the manufacturer’s protocol with proteinase K lysis and column-based purification. Negative (blank) and positive (known bacterial genomic DNA) controls were included to monitor contamination and extraction efficiency. DNA concentration and purity were verified prior to amplification. The V3–V4 hypervariable regions of the bacterial 16S rRNA gene were amplified with universal primers. The amplicons were purified and sequenced on the Illumina NovaSeq platform using paired-end chemistry. Raw sequences were processed through the QIIME2 [31] pipeline for quality filtering, denoising, and generation of amplicon sequence variants (ASVs). Taxonomic assignment was performed against the Silva database (Release 138, https://www.arb-silva.de/documentation/release-138/, accessed on 6 January 2026) to profile the bacterial community structure and identify potential pathogens within the lung tissue.

## 3. Results

### 3.1. Pathological Alterations in the Respiratory System

**Figure 1:**
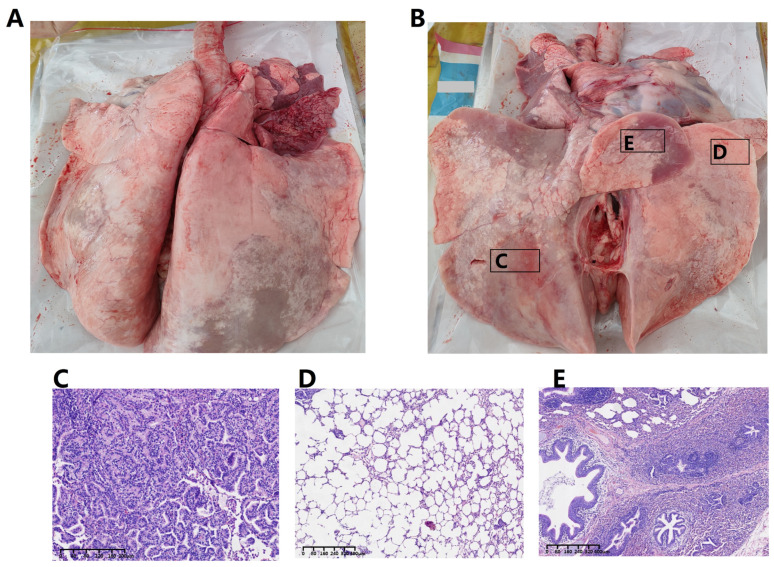
Pathological alterations in the lungs of a Hu sheep infected with *Mycoplasma ovipneumoniae* strain IM-DMQ. (**A**) Gross lesion showing caseous transformation and consolidation on the lung surface. (**B**) Gross lesion depicting severe fibrinous pleuritis and adhesion. (**C**) Histopathological section showing pulmonary adenoma with proliferative glandular epithelium arranged in duct-like configurations and amyloid deposition (H&E stain, purple). (**D**) Histopathological section showing compensatory emphysema characterized by expanded alveolar spaces (H&E stain, purple). (**E**) Histopathological section showing combined bronchopneumonia and interstitial pneumonia with marked peribronchial leukocyte infiltration (H&E stain, purple).

Postmortem evaluation revealed extensive pulmonary damage consistent with advanced mycoplasmal infection. Macroscopic inspection identified severe fibrinous pleuritis with significant adhesion formation between pulmonary and thoracic surfaces. The lung parenchyma showed diffuse consolidation and emphysematous changes, presenting the typical “white lung” pathology. Multifocal lesions included substantial consolidation in the right anterior, cardiac, and apical lobes, along with discrete mass formations distributed throughout all pulmonary lobes (Figure 1A,B).

**Figure 2:**
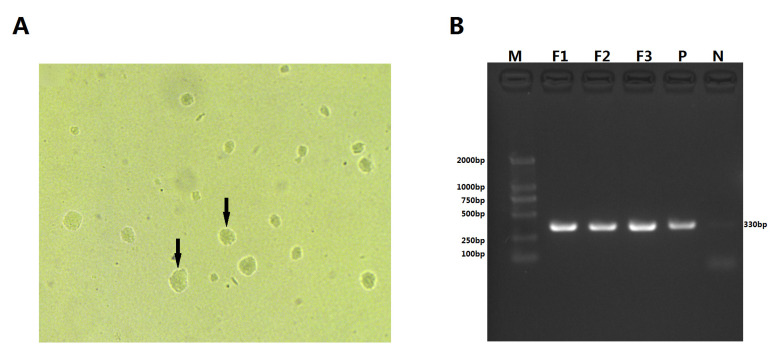
Isolation and PCR identification of *M. ovipneumoniae*. (**A**) Multi-colony of *M. ovipneumoniae* (40×) (The black arrow indicates a representative M. ovipneumoniae colony); (**B**) PCR confirmation of *M. ovipneumoniae*. Lane M: DNA marker DL2000. Lanes F1, F2, and F3: Genomic DNA from the *M. ovipneumoniae* isolate at three successive culture passages (F1 corresponds to the primary isolate IM-DMQ). Lane P: Positive control (DNA from a known *M. ovipneumoniae* strain). Lane N: Negative control (no template). The expected ~355 bp amplicon, specific to the *M. ovipneumoniae* 16S rRNA gene, is visible in lanes F1–F3 and P.

Histopathological examination distinguished three distinct pathological patterns: (1) Pulmonary adenoma exhibiting proliferative glandular epithelium arranged in duct-like configurations with associated amyloid deposition (Figure 1C); (2) compensatory emphysema characterized by expanded alveolar spaces with attenuated septal structures, without significant inflammatory infiltration (Figure 1D); and (3) combined bronchopneumonia and interstitial pneumonia displaying alveolar septal thickening, luminal constriction, and marked peribronchial leukocyte infiltration with purulent exudation (Figure 1E). It is important to note that proliferative adenomatous-like lesions, as observed here, represent an uncommon pathological presentation for *M. ovipneumoniae* infection. Their occurrence in this case prompted further microbial investigation but does not in itself establish etiology.

### 3.2. Isolation and Phenotypic Characterization of M. ovipneumoniae

Microbiological cultivation in modified KM2 medium successfully supported mycoplasma proliferation, as evidenced by characteristic metabolic color transition and turbidity development within 48 h. Primary isolation on solid medium yielded translucent pinpoint colonies after 36 h, which developed into distinctive “mulberry-like” colonial morphology upon maturation (Figure 2A). Molecular identification confirmed the isolate as *M. ovipneumoniae* through PCR amplification of the 16S rRNA gene (Figure 2B), demonstrating 99.71% sequence identity with reference strains. The isolate was designated *M. ovipneumoniae* strain IM-DMQ.

### 3.3. Genomic Characteristics of M. ovipneumoniae Strain IM-DMQ

Integrated assembly of long-read Nanopore (1.91 Gb clean data) and short-read Illumina (1.03 Gb clean data) sequences produced a complete circular genome of 1,039,804 bp with a GC content of 29.15%. Genomic annotation predicted 1529 genes, including 1493 protein-coding sequences, 30 tRNA genes, and single copies of 23S, 16S, and 5S rRNA genes (Table 1, Supplementary Figure S1). The genome contained diverse mobile genetic elements comprising 7 CRISPR arrays (Supplementary Table S1), 2 genomic islands, and 326 repetitive sequences categorized as SINE (4), LINE (6), and DNA transposons (1). Additionally, the assembly included a 1496-bp small closed circular DNA molecule. No significant homologous sequences were found for this element in public nucleotide databases, and its biological function requires further investigation (Supplementary Table S2).

### 3.4. Functional Genomic Characterization

**Figure 3:**
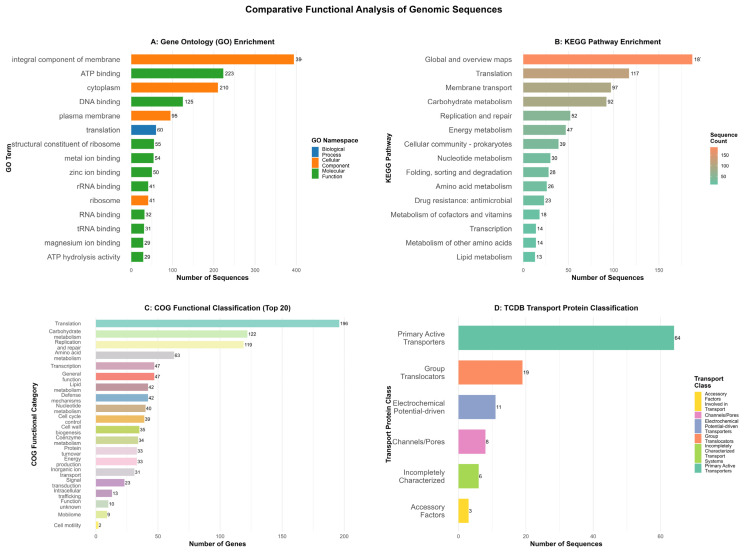
Functional annotation of the *M. ovipneumoniae* IM-DMQ genome. (**A**) Enriched Gene Ontology (GO) terms (Molecular Function category). (**B**) Distribution of Kyoto Encyclopedia of Genes and Genomes (KEGG) pathways. (**C**) Assignment of protein-coding sequences to Clusters of Orthologous Groups (COG) categories. (**D**) Classification of predicted transport systems according to the Transporter Classification Database (TCDB). Detailed gene lists corresponding to each annotation are provided in Supplementary Table S3.

Gene Ontology classification revealed significant enrichment in fundamental biological processes including cellular organization, metabolic regulation, and biosynthetic pathways (Figure 3A) (Supplementary Table S3). COG analysis indicated predominant representation in “Translation and ribosomal biogenesis” (24.1%), “Carbohydrate metabolism” (18.7%), and “DNA replication/repair” (15.3%) categories (Figure 3C). KEGG pathway mapping demonstrated primary involvement in metabolic (51.2%) and genetic information processing (28.7%) pathways (Figure 3B).

Specialized functional annotation identified membrane transport systems mainly composed of primary active transporters and group translocators (Figure 3D). Pathogen-host interaction analysis revealed potential virulence determinants including NifS [32] and MalF [33,34] homologs (Supplementary Table S4). Virulence factor screening identified conserved elongation factor Tu (98.2% identity) and pyruvate dehydrogenase (96.7% identity), while adhesion molecules showed moderate similarity (50–62%) to corresponding proteins in related mycoplasma species (Supplementary Table S5).

### 3.5. Phylogenomic and Structural Genomic Analysis

**Figure 4:**
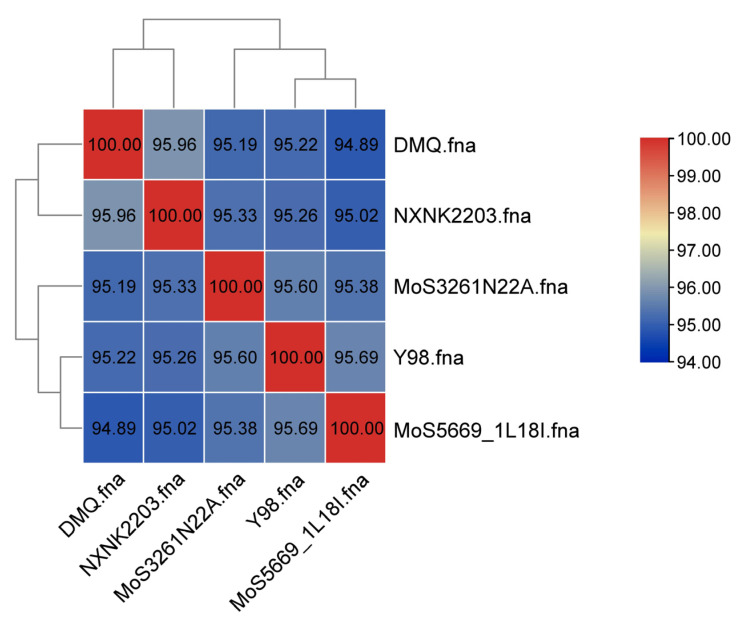
Heatmap of ANI analysis.

Average nucleotide identity (ANI) analysis demonstrated that IM-DMQ shares the closest genetic relationship with Chinese strains NXNK2203 (ANI: 98.3%) and Y98 (ANI: 96.5%), while showing greater evolutionary distance from the Icelandic strain MoS5669_1L18I (ANI: 94.7%) and Austrian strain MoS3261N22A (ANI: 95.2%) (Figure 4, Table 2). These ANI values confirm that IM-DMQ belongs to the same species as the Chinese isolates while exhibiting significant genomic divergence from European strains.

**Figure 5:**
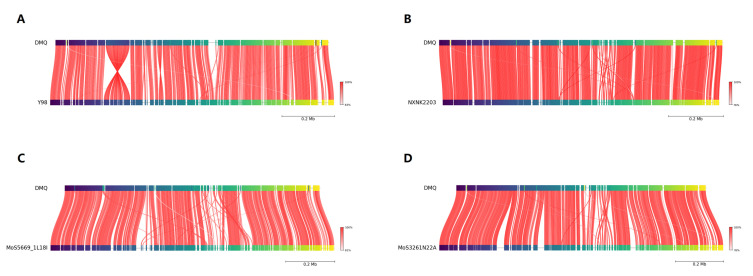
Conserved Regions of Two Genomes. The upper axis represents the DMQ genome, the lower axis represents the reference genome, the line between the upper and lower axes represents the corresponding position of the reference gene and the reference gene, and the color from red to white indicates the similarity from 100% to 0%. (**A**) Whole-genome sequence alignment between strain Y98 and the reference strain IM-DMQ, (**B**) Whole-genome sequence alignment between strain NXNK2203 and the reference strain IM-DMQ, (**C**) Whole-genome sequence alignment between strain MoS5669_1L18I and the reference strain IM-DMQ, (**D**) Whole-genome sequence alignment between strain MoS3261N22A and the reference strain IM-DMQ.

Comparative genomic examination further identified specific structural variations between IM-DMQ and reference strains. Relative to Y98, the IM-DMQ genome displayed several genomic rearrangements including large-scale inversions and positional relocation of gene clusters (Figure 5A). Notably, five unique genes were identified in IM-DMQ that were absent from the Y98 genome (Supplementary Data S1). In contrast, IM-DMQ and NXNK2203 strains exhibited strong genomic synteny without evidence of gene acquisition or loss, though minor positional variations were observed (Figure 5B).

Discernible structural differences were evident in comparison with geographically distant isolates. The Icelandic strain MoS5669_1L18I showed multiple gene position alterations (Figure 5C), while comparison with MoS3261N22A (which has a complete, circular genome of 1,182,340 bp) revealed both positional changes and some gene content differences, with 14 genes present in IM-DMQ but absent in the Austrian strain (Figure 5D, Supplementary Data S2). Overall, these structural variations occur within a conserved genomic backbone, as reflected by the high pairwise ANI values (>94.89%) shared among all compared strains.

### 3.6. Microbial Community Analysis of the Ovine Lung Tissue Sample

To directly assess the microbial composition within the affected lung, 16S rRNA gene amplicon sequencing was performed on the same lung tissue sample used for pathogen isolation. Genus-level community profiling (Supplementary Figure S2) revealed a complex bacterial assemblage. Notably, sequences classified as Mycoplasma constituted the most abundant genus, with a relative abundance of 16.17%. This finding provides direct molecular confirmation of a substantial Mo load in the primary tissue, correlating with our successful isolation and identification of the *M. ovipneumoniae* strain IM-DMQ. Furthermore, we concurrently detected sequences belonging to several other known respiratory pathogens, including Pasteurella (2.45%), Mannheimia (2.22%), and Streptococcus (1.87%). All detected genera with potential pathogenic relevance and their corresponding relative abundances are detailed in Supplementary Table S8. This analysis provides molecular evidence for the direct co-detection of multiple pathogens in the original sample and establishes a basis for discussing the potential role of polymicrobial infection in the following section.

## 4. Discussion

### 4.1. Potential Link Between M. ovipneumoniae Infection and Ovine Pulmonary Adenomatosis

*Mycoplasma ovipneumoniae* strain IM-DMQ was isolated from the lung of a Hu sheep that presented with severe respiratory disease and uncommon pulmonary adenomatous-like proliferative lesions. It is critical to emphasize that this study primarily reports the genomic characterization of this bacterial isolate. While the co-occurrence of this pathogen with atypical pathology is notable, our investigation—based on a single clinical case—was not designed to establish etiology. The necropsy and histopathological assessment were performed by trained veterinary pathologists; however, potential alternative or contributing factors, including other infectious agents or environmental components, were not systematically excluded. Therefore, no causal relationship between *M. ovipneumoniae* IM-DMQ and the observed proliferative lesions can be inferred from the present data.

The isolation of this strain from such a lesion, however, provides a relevant context for discussing potential pathogenic mechanisms. In the broader literature, certain mycoplasma species have been implicated in processes related to chronic inflammation and cellular proliferation [35,36,37,38]. For instance, secreted mycoplasmal proteins such as DnaK have been shown to interfere with host cell regulatory pathways, including DNA repair and tumor suppression mechanisms in other systems [9,39,40]. Furthermore, chronic mycoplasma infections have been associated with sustained inflammation, genomic instability, and modulation of apoptosis, which may collectively influence tissue remodeling [40,41,42,43].

Drawing parallels from these studies in different biological contexts, we hypothesize that chronic *M. ovipneumoniae* infection could potentially act as a contributory factor in the development or exacerbation of complex proliferative pulmonary responses in sheep. This remains a speculative association grounded in mechanistic observations from related pathogens, not a conclusion derived from our data. The exact role, if any, of *M. ovipneumoniae* in ovine pulmonary adenomatosis or similar proliferative conditions necessitates rigorous future investigation through controlled epidemiological studies, experimental infection models, and molecular pathogenesis research.

### 4.2. Genomic Architecture and Functional Profile of IM-DMQ

We present the first complete genome sequence of an *M. ovipneumoniae* strain (IM-DMQ) obtained from Hu sheep in Inner Mongolia. The IM-DMQ genome consists of a 1,039,804 bp circular chromosome with a GC content of 29.15%, encoding 1529 genes, among which 1493 are predicted CDSs, along with 30 tRNAs and 3 rRNAs. Gene ontology analysis indicated significant enrichment in core biological functions, including ribosomal biogenesis, carbohydrate metabolism, and nucleic acid replication and repair, which are characteristics reflective of the host-adapted and fastidious metabolism typical of mycoplasmas [3,44].

Genomic analysis further identified 7 CRISPR loci, 2 genomic islands, and 326 repetitive elements (Supplementary Tables S6 and S7). These features are frequently linked to bacterial genome evolution, environmental adaptation, and virulence modulation [45,46]. The presence of an intact CRISPR-Cas system implies a capacity for defense against mobile genetic elements, which may influence strain-specific pathogenicity [47].

### 4.3. Putative Virulence Determinants and Host–Pathogen Interplay

Using the Pathogen–Host Interaction (PHI) database, we annotated several IM-DMQ genes potentially involved in virulence regulation, such as NifS and MalF. In related pathogens, homologs of these genes contribute to attenuated pathogenicity [48], indicating a possible role in Mo host adaptation. Virulence factor screening also identified highly conserved candidates including elongation factor Tu (Tuf) and pyruvate dehydrogenase subunit beta (PdhB), both exhibiting >90% sequence identity with established virulence-associated proteins [49,50]. Although adhesion factors such as P97 and P102 showed limited sequence similarity (∼50–62%) to those in Mycoplasma suis, their conservation suggests a possible role in host cell attachment [51,52,53].

Functional validation of these genes remains a critical next step. The development of genetic manipulation tools for Mo, including gene knockout and complementation systems [54,55,56], will be essential for elucidating molecular mechanisms of infection and informing future vaccine and diagnostic development.

### 4.4. Genomic Comparison and Phylogenetic Context

Average nucleotide identity (ANI) analysis revealed that IM-DMQ is most closely related to the Chinese strain NXNK2203 (ANI > 95%), whereas it shares lower identity (94.89%) with an Icelandic isolate (MoS5669_1L18I) [57]. This pattern of genetic similarity, observed across intercontinental distances, could suggest that geographic separation may be one factor influencing the population structure of *M. ovipneumoniae*. This observation aligns with a recent comparative genomic study that identified population structure and signals of adaptive variation correlated with geographic origin in a broad collection of strains [13]. It is noteworthy, however, that other studies focusing on regional scales have reported limited correlation between geography and genetic variation, highlighting the potential influence of local transmission dynamics [58]. Collectively, these findings indicate that the genetic landscape of *M. ovipneumoniae* is likely shaped by multiple factors, including geographic separation, host adaptation, and local epidemiological patterns.

Structural genomic comparisons revealed rearrangements, inversions, and unique genomic regions between IM-DMQ and other strains, which may account for variations in host specificity and virulence [13]. Notably, IM-DMQ contains 14 open reading frames not found in the Austrian reference strain MoS3261N22A. Investigating the functions of these unique genes may provide insights into the adaptive evolution and pathogenic mechanisms of *M. ovipneumoniae* in regional sheep populations [4,59].

### 4.5. Etiological Considerations from the Microbial Community Data

The microbial community analysis provides direct molecular evidence for a polymicrobial infection in the sampled lung. The high abundance of Mycoplasma supports its primary role, while the co-presence of Pasteurella and Mannheimia suggests a probable severe secondary bacterial pneumonia, which is a common sequela of mycoplasmal infection [60,61,62]. This context offers a plausible explanation for the acute, fatal outcome. A critical limitation of this ancillary analysis must be noted. The 16S rRNA gene sequencing approach is inherently designed for bacterial detection and cannot identify viral pathogens, such as the ovine pulmonary adenomatosis virus (JSRV). Therefore, the potential role of JSRV in initiating the adenomatous-like proliferative lesions remains unresolved and requires exclusion or confirmation in future studies using specific assays like PCR or immunohistochemistry. Furthermore, relative abundance derived from sequencing is an ecological metric; it does not directly equate to the absolute quantity or the in vivo pathogenic activity of the detected organisms. Nonetheless, within this complex background, the isolation and genomic characterization of the IM-DMQ strain establish a valuable resource. Its unique genomic features may inform future studies on *M. ovipneumoniae* adaptation and pathogenesis in polymicrobial settings.

## 5. Conclusions

This research reports the successful isolation and comprehensive genomic analysis of *Mycoplasma ovipneumoniae* strain IM-DMQ from pulmonary tissues of Hu sheep presenting adenomatous-like lesions in Inner Mongolia. Genomic characterization revealed characteristic metabolic limitations typical of mycoplasma species, along with the identification of potential virulence factors implicated in host-pathogen interactions. Phylogenetic comparisons demonstrated significant genomic homology with Chinese isolates compared to European strains, indicating potential geographical evolutionary patterns. Importantly, microbial community data from the infected tissue suggested a polymicrobial infection scenario, which plausibly contributed to the fatal outcome. While the exact etiological agent of the adenomatous-like lesions remains undefined and warrants future investigation, this study establishes the first complete genomic resource for an *M. ovipneumoniae* strain (IM-DMQ) from Inner Mongolia, characterized within its clinical and microbial context. The genomic resources established in this study provide crucial insights into the molecular mechanisms of *M. ovipneumoniae* pathogenicity and form a foundation for developing targeted disease control strategies. Future studies should focus on functional validation of the identified virulence factors to elucidate their specific roles in disease pathogenesis.

## Figures and Tables

**Table 1:** Summary of genomic features and gene prediction results for *Mycoplasma ovipneumoniae* strain IM-DMQ.

Type	Number	Total_len	Average_len	Percentage of Genome (%)
Gene	1529	682,833	447	65.58
CDS	1493	675,450	452	64.87
tRNA	30	2367	79	0.23
23S rRNA	1	2882	2882	0.28
16S rRNA	1	1527	1527	0.15
5S rRNA	1	69	69	0.01
tmRNA	0	0	0	0.00
misc_RNA	3	538	179	0.05

Type: the predicted gene type; Number: the number of genes predicted; Total_len: predicted gene length (bp); Average_len: predicted average gene length (bp); Percentage of genome (%): Percentage of the genome length.

**Table 2:** Basic information on each strain of the Mo cluster.

Strain	Accession Number	Years of Isolation	Location of Isolation	Size (bp)	G + C %
Y98	CP118522.1	1971	Australia	1,081,520	29
MoS5669_1L18I	CP134945.1	2018	Iceland	1,158,390	29
IM-DMQ	Not yet uploaded	2019	Inner Mongolia of China	1,039,804	29.15
NXNK2203	CP124621.1	2022	NingXia of China	1,014,835	29
MoS3261N22A	CP134944.1	2022	Austria	1,182,340	29

## Data Availability

The original contributions presented in the study are included in the article/Supplementary Materials. Further inquiries can be directed to the corresponding authors.

## Supplementary Materials

The following supporting information can be downloaded at: https://www.mdpi.com/article/10.3390/vetsci13010079/s1, Supplementary Figure S1. Whole-genome circle map of Mo IM-DMQ; Supplementary Figure S2. Top15_genera_barplot; Supplementary Table S1. Clustered Regularly Interspaced Palindromic Repeats (CRISPR); Supplementary Table S2. Statistical Results of Genome Assembly; Supplementary Table S3. Gene annotation were performed in different databases; Supplementary Table S4. Potential proteins for Pathogen Host Interactions; Supplementary Table S5. Potential virulence proteins; Supplementary Table S6. Predicted genomic islands; Supplementary Table S7. Repetitive elements statistics; Supplementary Table S8. Genus_abund_M12; Supplementary_Data_S1_Unique_genes_in_IM-DMQ_vs_Y98; Supplementary_Data_S2_Unique_genes_in_IM-DMQ_vs_MoS3261N22A.
