# Supplementary material for: Genomic Characterization of a Mycoplasma ovipneumoniae Strain from Hu Sheep in Inner Mongolia, China
Source: Vet Sci. 2026 Jan 13;13(1):79. doi: 10.3390/vetsci13010079 (PMC12846336; doi:10.3390/vetsci13010079)
Supplement: Supplementary file 1 [file vetsci-13-00079-s001.zip › Supplementary Figure S1. Whole-genome circle map of Mo IM-DMQ.pdf]

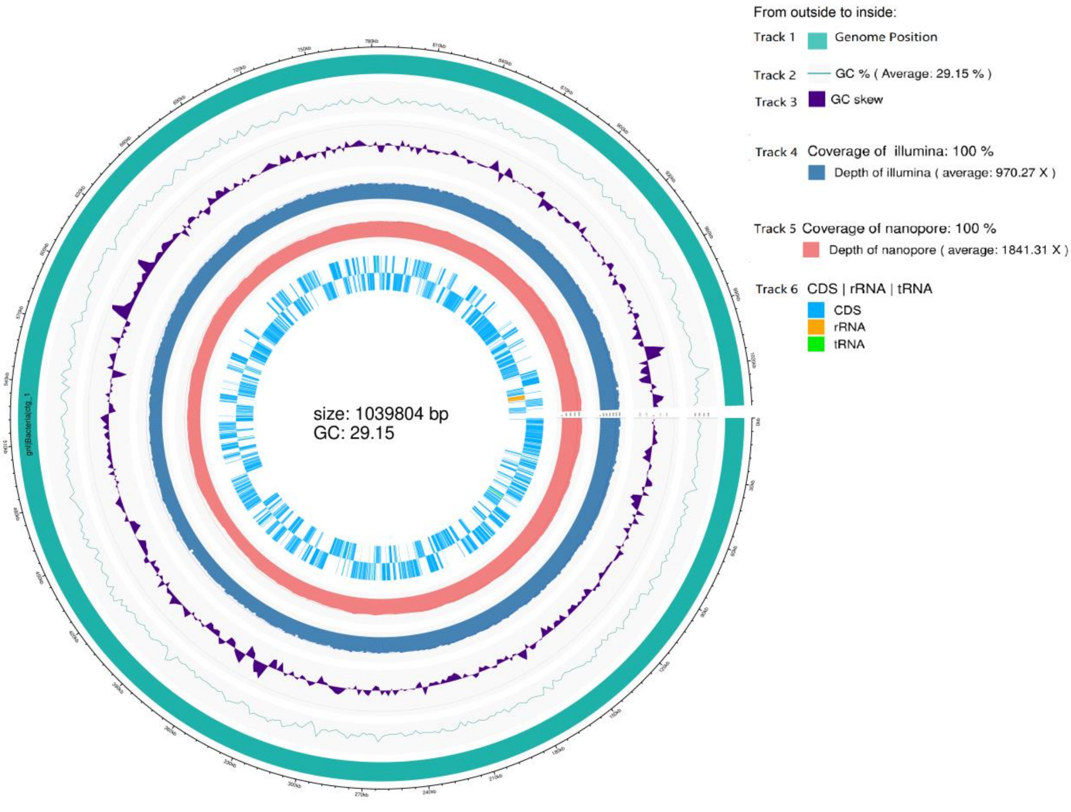

Supplementary Figure S1. Whole-genome circle map of Mo IM-DMQ. The circles' diagram from outside to inside is genomic sequence information, plus and minus strand COG, GC content curve of the genomic sequence, GC skew curve of the genomic sequence, information on second-generation sequencing depth and coverage, information on the depth and coverage of the three generations of sequencing, and the coding regions of genes (CDS) as well as non-coding RNA regions (rRNA, tRNA) in the reference genome. The window and step size are both 2000bp.
