# Supplementary figures and images for: Genomic Characterization of a Mycoplasma ovipneumoniae Strain from Hu Sheep in Inner Mongolia, China
Source: Vet Sci. 2026 Jan 13;13(1):79. doi: 10.3390/vetsci13010079 (PMC12846336; doi:10.3390/vetsci13010079)

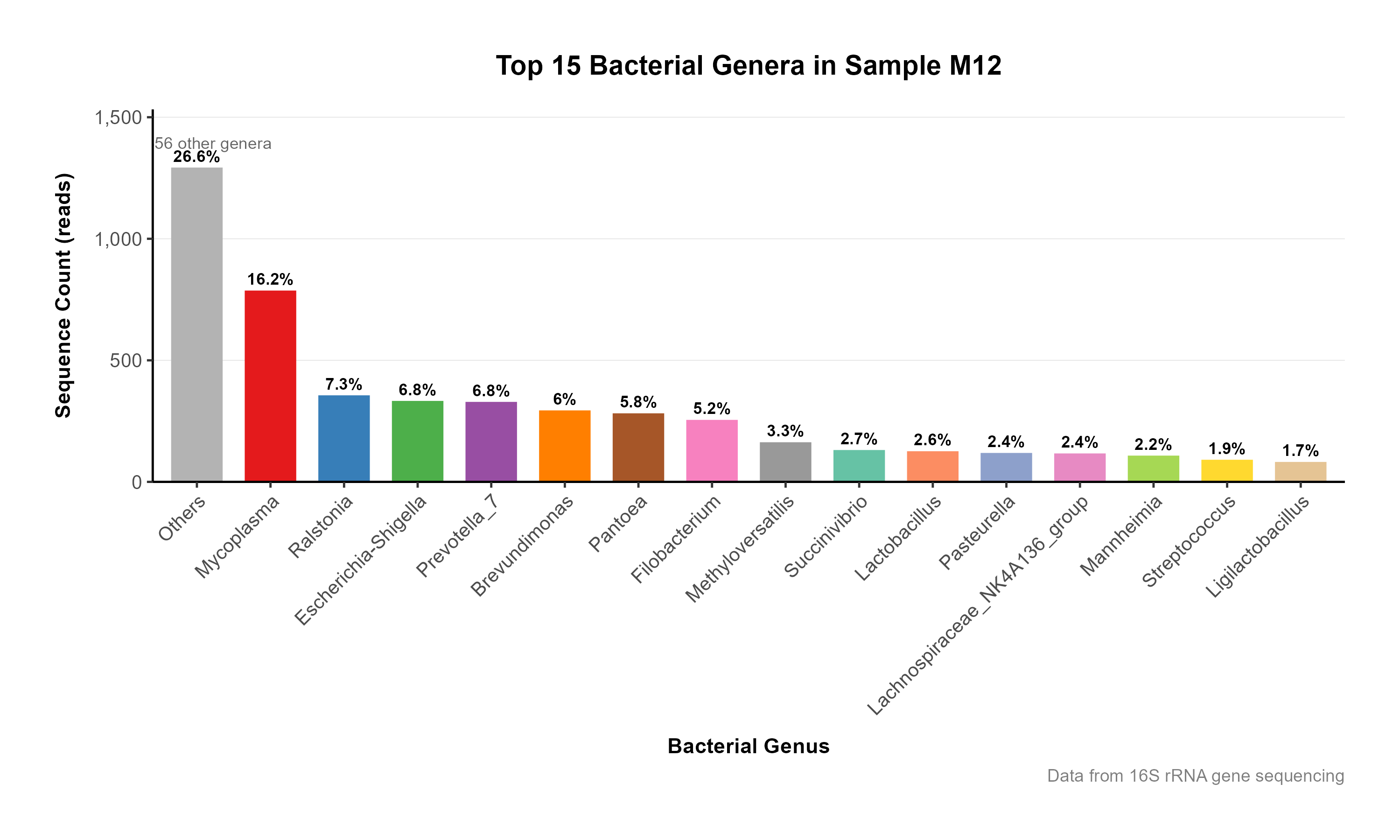

Supplement: Supplementary file 1 [file vetsci-13-00079-s001.zip › Supplementary Figure S2 Top15_genera_barplot.png]
